# Population levels of wellbeing and the association with social capital

**DOI:** 10.1186/s40359-017-0193-0

**Published:** 2017-07-03

**Authors:** A. W. Taylor, G. Kelly, E. Dal Grande, D. Kelly, T. Marin, N. Hey, K. J. Burke, J. Licinio

**Affiliations:** 1 Population Research & Outcome Studies, Discipline of Medicine, The University of Adelaide, Adelaide, South Australia Australia; 2 Wellbeing and Resilience Centre, South Australian Health and Medical Research Institute (SAHMRI), Adelaide, Australia; 3 What Works Centre for Wellbeing, London, UK; 4 CQUniversity, Appleton Institute, School of Human, Health & Social Sciences, Wayville, South Australia Australia

**Keywords:** Wellbeing, Social capital, Australia, Population

## Abstract

**Background:**

This research investigates wellbeing at the population level across demographic, social and health indicators and assesses the association between wellbeing and social capital.

**Method:**

Data from a South Australian monthly chronic disease/risk factor surveillance system of randomly selected adults (mean age 48.7 years; range 16–99) from 2014/5 (*n* = 5551) were used. Univariable analyses compared wellbeing/social capital indicators, socio-demographic, risk factors and chronic conditions. Multi-nominal logistic regression modelling, adjusting for multiple covariates was used to simultaneously estimate odds ratios for good wellbeing (reference category) versus neither good nor poor, and good wellbeing versus poor wellbeing.

**Results:**

48.6% were male, mean age 48.7 (sd 18.3), 54.3% scored well on all four of the wellbeing indicators, and positive social capital indicators ranged from 93.1% for safety to 50.8% for control over decisions. The higher level of social capital corresponded with the good wellbeing category. Modeling showed higher odds ratios for all social capital variables for the lowest level of wellbeing. These higher odds ratios remained after adjusting for confounders.

**Conclusions:**

The relationship between wellbeing, resilience and social capital highlights areas for increased policy focus.

## Background

Wellbeing and social capital are two dissociable but connected measureable attributes of individuals and communities. Understanding the role of social capital in building and strengthening wellbeing at the population level is an important consideration when aiming for best possible experience and functioning of the population [1].

The benefits of positive wellbeing have been shown to be associated with improved mental and physical health and overall enhanced quality of life [2–4]. An important notion within the positive wellbeing concept is resilience, broadly defined as the ability to bounce-back from negative events [4, 5]. Resilience is also defined as the ability to capitalize on opportunity [6]. Large-scale/small-time, minor/major adverse events or catastrophes occur in our daily lives and individuals and populations also have to deal with stress in times of economic downturns or social turmoil [7]. Developing personal skills to overcome negative events in times of stress by increasing levels of resilience can assist individuals and communities to succeed in an environment that can be typified by change, insecurity and volatility [8]. Dynamic economic circumstances also require a flexible approach to employment and the ability to retrain or seize opportunity.

Social capital, broadly defined as connectedness within and between populations, and the quality and quantity of social relations within that population [9], is a multi-disciplinary and multi-faceted, well researched area that encompasses social networks, trust, reciprocity and support [4, 9]. ‘Bonding’ social capital is often used to describe the social relationship between individuals while ‘bridging’ social capital is seen as that between groups [4]. Although the definition of social capital is contested [7], it is acknowledged that social capital operating at both the micro and macro levels of society is related to health outcomes [1, 9–11]. The debate regarding definition and measurement of social capital is not the focus of this paper; rather we aim to assess the association between social capital and wellbeing and resilience to provide additional explanatory factors [12].

While many governments have incorporated goals and targets into their portfolios often these are dominated by economic and demographic metrics. South Australia has embraced a state-wide approach to building, embedding and researching wellbeing and resilience. This strategy aims to increase the state’s population level of positive wellbeing with an overall aim of assisting the society to thrive by measuring and building its level of resilience. As such, initiatives within schools, workplaces and communities have been introduced. Questions to assess the level of the wellbeing of the population have been incorporated into the South Australian government’s monthly risk factor and chronic disease surveillance system [13] so that the subjective wellbeing at population and sub-population level, can be monitored over time. As argued by others, measuring and assessing wellbeing is crucial for assessing the effectiveness of health promotion and population health wellness-orientated endeavours and initiatives [14, 15].

Research has shown that social capital is an important aspect of resilience following major disasters or large scale crisis [7]. Exploring the relationship between social capital, wellbeing and resilience in a community without a natural disaster or large scale acute event, provides policy makers and decision makers evidence, and an additional tool, to effect change to assist in the development of policy interventions to increase general wellbeing in the community [16].

Our aim therefore is to detail the levels of wellbeing at the population level in South Australia by a range of demographic, social, economic and health indicators and to assess the association between wellbeing and measures of social capital using models with the data adjusted for known confounders.

## Methods

The data for these analyses were obtained from the South Australian Monitoring and Surveillance System (SAMSS), a monthly chronic disease and risk factor surveillance system of randomly selected persons, established in July 2002 [17]. All households in SA with a telephone number listed in the Electronic White Pages (EWP) are eligible for selection in the sample. A letter introducing SAMSS is sent to the household of each selected telephone number. Within each household the person who had a birthday last is selected for interview. There is no replacement for non-contactable persons. Data are collected by a contracted agency using Computer Assisted Telephone Interviewing (CATI) and interviews are conducted in English. Informed consent was obtained before the start of the interview. Detailed SAMSS methodology has been published elsewhere [13, 17].

Although SAMSS data have been collected since July 2002, questions on wellbeing were included from January 2014. Analysis was limited to participants aged 18 years and over (*n* = 5551). The monthly response rate (RR1) of SAMSS for this period ranged from 54.0 to 61.5 (mean = 56.9) [18].

Demographic covariate variables included in the analyses were sex, age, area of residence (metropolitan, rural, remote), country of birth, marital status, highest educational attainment and household money situation. Co-morbidity conditions included self-reported, medically confirmed diabetes, current asthma, cardio-vascular disease (heart attack, angina, heart disease and/or stroke), arthritis and osteoporosis. Self-reported health risk factor data included physical activity (derived on the amount of walking and moderate and vigorous activity in a 1 week period) [19], body mass index (BMI) which was derived from self-reported weight and height and recoded into four categories (underweight, normal weight, overweight and obese) [20], current smoking status, alcohol risk (derived from the number of alcoholic drinks per day and the number of times per week alcohol was consumed) [21], and inadequate daily consumption of vegetables and fruit (sufficient vegetables = 2+ per day; sufficient fruit = 1+ per day) [22].

The four wellbeing questions were sourced from the UK Office for National Statistics [23] and were 1) Life satisfaction (Overall, how satisfied are you with your life nowadays?); 2) Worthwhile (Overall, to what extent do you feel the things you do in your life are worthwhile?); 3) Happy yesterday (Overall, how happy did you feel yesterday?); and 4) Anxious yesterday (Overall, how anxious did you feel yesterday?). Each was scored on a scale of 0 to 10 where 0 meant “not at all” and 10 meant “completely”. To score well on all four measures (indicating good wellbeing) respondents had to, for Life satisfaction, Worthwhile, and Happy yesterday, score 8 to 10 and for Anxious yesterday score 0 to 2 [23, 24].

Four questions were asked as surrogate measures of social capital. They were ‘overall, do you feel that your neighbourhood is a safe place’ (yes, no); ‘do you think that in this neighbourhood people generally trust one another’ (yes, no); ‘do you feel safe in your home’ (all of the time, most of the time, some of the time, none of the time) and ‘I have control over the decisions that affect my life’ (strongly agree, agree, neutral/don’t know, disagree, strongly disagree).

SAMSS data were weighted each month by age, sex, area and probability of selection in the household to estimated resident population data of the most recent Australian Bureau of Statistics Census or estimated residential population data, so that the results were representative of the South Australian population. Probability of selection in the household was calculated on the number of eligible people in the household and the number of listings in the EWP. The weights reflect unequal sample inclusion probabilities and compensate for differential non-response.

Analyses were conducted using SPSS Version 20 and Stata Version 13. Initial analyses included frequencies for the four individual and overall wellbeing (good, neither good nor poor, and poor) and social capital indicators. Univariable analyses using chi-square tests compared the overall wellbeing and the four social capital indicators, socio-demographic, risk factors and chronic conditions. Factors associated with neither good nor poor and low levels of wellbeing including risk factors, socio-economic and socio-demographic variables and concepts of social capital were assessed using multi-nominal logistic regression modelling using all three levels of wellbeing with good wellbeing as the reference category adjusting for multiple covariates. Multi-nominal logistic regression was used to simultaneously estimate odds ratios for two different comparisons: good wellbeing (reference category) versus neither good nor poor, and good wellbeing versus poor wellbeing. Model 1 adjusted for age and sex, and model 2 adjusted for age, sex, country of birth, area of residence, educational attainment, marital status, money situation and the number of adults in the household. The unadjusted model is also presented.

## Results

Of the total sample 48.6% were male. Mean age was 48.7 (standard deviation 18.3) years (median 48 years). Table 1 highlights the distribution of the four individual wellbeing questions, a summary of the proportion scoring well or badly or neither on all measures, and a distribution of the six social capital related variables. In total, 54.3% of the South Australian adult population scored well on all four of the wellbeing indicators, while the range of positive responses to the social capital indicators ranged from 93.1% for safety to 50.8% for control over decisions.

**Table 1:** Prevalence of four individual wellbeing indicators and social capital indicators, aged 18 years and over by year, 2014–15

	n	% (95% CI)
INDIVIDUAL WELLBEING INDICATORS		
Life satisfaction		
Very low (0–4)	173	3.1 (2.7–3.6)
Low (5–6)	515	9.3 (8.5–10.1)
Medium (7–8)	2633	47.4 (46.1–48.8)
High (9–10)	2186	39.4 (38.1–40.7)
Don’t know, refused	43	0.8 (0.6–1.0)
Worthwhile		
Very low (0–4)	108	1.9 (1.6–2.3)
Low (5–6)	430	7.7 (7.1–8.5)
Medium (7–8)	2374	42.8 (41.5–44.1)
High (9–10)	2563	46.2 (44.9–47.5)
Don’t know, refused	75	1.3 (1.1–1.7)
Happy yesterday		
Very low (0–4)	256	4.6 (4.1–5.2)
Low (5–6)	469	8.5 (7.8–9.2)
Medium (7–8)	2021	36.4 (35.2–37.7)
High (9–10)	2772	49.9 (48.6–51.2)
Don’t know, refused	32	0.6 (0.4–0.8)
Anxious yesterday		
Very high (6–10)	484	8.7 (8.0–9.5)
High (4–5)	422	7.6 (6.9–8.3)
Medium (2–3)	694	12.5 (11.7–13.4)
Low (0–1)	3917	70.6 (69.3–71.7)
Don’t know, refused	34	0.6 (0.4–0.9)
Total	5551	100.0
Overall Wellbeing (composite score)		
Scoring well on all four measures	2968	54.3 (53.0–55.6)
Scoring neither well nor badly	1764	32.3 (31.0–33.5)
Scoring badly on at least one measure	733	13.4 (12.5–14.3)
Total	5464	100.0
SOCIAL CAPITAL INDICATORS		
Overall, do you feel that your neighbourhood is a safe place?		
Yes	5167	93.1 (92.0–94.1)
No, don’t know	383	6.9 (5.9–8.0)
Do you think that in this neighbourhood people generally trust one another?		
Yes	4379	78.9 (77.2–80.5)
No, don’t know	1172	21.1 (19.5–22.8)
Do you feel safe in your home?		
All of the time	4252	76.6 (74.9–78.2)
Most, some or none of the time	1299	23.4 (21.8–25.1)
Do you agree or disagree with the following statement. I have control over decisions that affect my life		
Strongly agree, agree	5239	94.4 (93.4–95.2)
Neutral, don’t know	105	1.9 (1.5–2.5)
Disagree, strongly disagree	206	3.7 (3.0–4.6)
Total	5551	100.0

The univariable distribution of the social capital indicators across the levels of wellbeing is highlighted in Table 2. In all instances the higher level of social capital corresponded with the good wellbeing category.

**Table 2:** Univariable analyses of overall wellbeing by social capital indicators

	Total	Good wellbeing		Scoring neither well or badly		Poor wellbeing		*P* value
	N	n	% (95% CI)	n	% (95% CI)	n	% (95% CI)	
Feel that your neighbourhood is a safe place								
Yes	5087	2849	56.0 (54.0–58.0)	1597	31.4 (29.5–33.3)	642	12.6 (11.2–14.2)	<0.001
No, don’t know, not sure	377	119	31.5 (25.0–38.8)	167	44.3 (36.5–52.4)	91	24.2 (18.2–31.4)	
Neighbourhood people generally trust one another								
Yes	4333	2499	57.7 (55.5–59.8)	1332	30.7 (28.8–32.8)	502	11.6 (10.2–13.2)	<0.001
No, don’t know, not sure	1131	469	41.5 (37.2–45.8)	431	38.1 (33.9–42.6)	231	20.4 (16.5–25.0)	
Feel safe in your home								
All of the time	4185	2465	58.9 (56.6–61.1)	1234	29.5 (27.5–31.5)	487	11.6 (10.1–13.4)	<0.001
Most, some or none of the time	1278	503	39.3 (35.5–43.4)	530	41.5 (37.4–45.6)	246	19.2 (16.1–22.8)	
Control over decisions affect life								
Agree	5172	2902	56.1 (54.1–58.1)	1650	31.9 (30.0–33.8)	620	12.0 (10.6–13.5)	<0.001
Neutral	98	30	31.2 (19.8–45.4)	40	40.7 (28.2–54.6)	27	28.1 (17.6–41.7)	
Disagree	194	35	17.8 (11.5–26.6)	74	38.1 (28.8–48.4)	86	44.1 (33.7–55.0)	
OVERALL	5464	2968	54.3 (52.3–56.3)	1764	32.3 (30.5–34.2)	733	13.4 (12.0–15.0)	

Table 3 highlights the relationship between relevant covariates and the three levels of wellbeing with all socio-demographic associations having a *p* value of <0.05 except country of birth and education level. Females, older persons and those who could save had higher estimates of good wellbeing. Higher levels of poor wellbeing were seen for younger respondents, those living in the metropolitan area, the never married and those unable to save.

**Table 3:** Univariable analyses of overall wellbeing and covariates (socio-demographic)

	Total	Good wellbeing		Scoring neither well or badly		Poor wellbeing		*P* value
	n	n	% (95% CI)	n	% (95% CI)	n	% (95% CI)	
COVARIATES								
Sex								
Male	2653	1357	51.2 (48.1–54.2)	923	34.8 (31.9–37.8)	373	14.1 (11.7–16.7)	0.014
Female	2811	1610	57.3 (54.8–59.7)	841	29.9 (27.7–32.2)	360	12.8 (11.2–14.6)	
Age group								
18–24	551	230	41.8 (35.8–48.1)	226	40.9 (34.9–47.3)	95	17.2 (13.2–22.3)	<0.001
25–34	910	421	46.3 (38.8–53.9)	296	32.5 (26.1–39.6)	193	21.2 (15.4–28.5)	
35–44	972	521	53.6 (48.4–58.7)	332	34.1 (29.4–39.2)	119	12.3 (9.3–16.1)	
45–54	1005	500	49.7 (45.4–54.0)	365	36.4 (32.2–40.8)	140	13.9 (11.3–17.0)	
55–64	897	542	60.4 (57.6–63.3)	257	28.6 (26.1–31.4)	98	10.9 (9.3–12.8)	
65–74	595	410	68.9 (66.3–71.3)	135	22.7 (20.5–25.1)	50	8.4 (7.0–9.9)	
75+	535	344	64.3 (61.4–67.1)	153	28.6 (26.0–31.5)	38	7.1 (5.7–8.7)	
Area of residence								
Metropolitan area	3985	2103	52.8 (50.3–55.2)	1304	32.7 (30.5–35.0)	577	14.5 (12.7–16.5)	0.008
Rural Centres	1409	825	58.6 (55.3–61.7)	435	30.8 (27.8–34.1)	149	10.6 (8.9–12.6)	
Remote Areas	70	39	55.7 (42.7–68.0)	25	35.7 (24.4–48.8)	6	8.6 (3.7–18.5)	
Marital status								
Married/De facto	3593	2144	59.7 (57.2–62.1)	1058	29.4 (27.3–31.7)	391	10.9 (9.3–12.8)	<0.001
Separated/Divorced	368	170	46.1 (41.1–51.2)	142	38.7 (33.7–43.9)	56	15.2 (12.0–19.1)	
Widowed	268	157	58.6 (54.9–62.3)	84	31.4 (28.0–35.0)	27	10 (8.1–12.3)	
Never married	1227	493	40.2 (35.4–45.1)	478	39 (34.4–43.8)	256	20.9 (17.2–25.1)	
Country of birth								
Australia	4279	2344	54.8 (52.6–57.0)	1363	31.9 (29.8–33.9)	572	13.4 (11.9–15.1)	0.481
UK and Ireland	554	307	55.5 (50.3–60.6)	175	31.7 (27.0–36.8)	71	12.8 (9.6–16.8)	
Other	630	316	50.2 (43.3–57.0)	225	35.8 (29.4–42.7)	88	14 (8.9–21.4)	
Educational attainment								
Up to secondary	2386	1250	52.4 (49.7–55.1)	790	33.1 (30.5–35.8)	346	14.5 (12.6–16.7)	0.156
Trade, Apprenticeship, Certificate, Diploma	1662	935	56.3 (52.6–59.9)	493	29.7 (26.5–33.0)	233	14 (11.6–17.0)	
Degree or higher	1411	778	55.2 (50.8–59.5)	480	34 (30.1–38.3)	152	10.8 (7.8–14.7)	
Number of adults								
1	698	338	48.3 (45.3–51.4)	247	35.4 (32.4–38.4)	114	16.3 (13.8–19.2)	<0.001
2	2970	1746	58.8 (56.3–61.3)	892	30.0 (27.7–32.5)	332	11.2 (9.7–12.9)	
3 or more	1796	884	49.2 (45.1–53.4)	625	34.8 (31.1–38.8)	287	16.0 (12.8–19.7)	
Household money situation								
Spending more than getting to some money left but spend it	1268	484	38.2 (34.5–42.0)	527	41.6 (37.7–45.6)	257	20.3 (17.4–23.5)	<0.001
Save a bit to save a lot	3903	2337	59.9 (57.5–62.2)	1126	28.8 (26.8–31.0)	440	11.3 (9.6–13.2)	
Not stated	293	147	50.1 (42.2–57.9)	111	37.8 (30.2–46.1)	36	12.1 (7.6–18.9)	
OVERALL	5464	2968	54.3 (52.3–56.3)	1764	32.3 (30.5–34.2)	733	13.4 (12.0–15.0)	

Table 4 highlights the relationship between chronic conditions, risk factors and wellbeing. All risk factors had a relationship except BMI. In terms of chronic conditions the only relationship was between current asthma and wellbeing.

**Table 4:** Univariable analyses of overall wellbeing and covariates (health-related variables)

	Total	Good wellbeing		Scoring neither well or badly		Poor wellbeing		*P* value
	n	n	% (95% CI)	n	% (95% CI)	n	% (95% CI)	
COVARIATES								
CHRONIC CONDITIONS								
Current asthma	762	347	45.5 (40.3–50.9)	255	33.5 (28.6–38.9)	160	20.9 (16.3–26.5)	<0.001
Arthritis	1147	610	53.2 (50.1–56.2)	376	32.8 (29.9–35.8)	161	14.0 (11.8–16.6)	0.763
Osteoporosis	238	133	55.8 (50.3–61.2)	66	27.8 (23.6–32.5)	39	16.3 (12.2–21.5)	0.163
Diabetes	443	225	50.7 (45.3–56.1)	143	32.3 (27.5–37.5)	75	17.0 (12.7–22.4)	0.180
CVD	393	207	52.6 (47.6–57.5)	124	31.5 (27.5–35.8)	63	15.9 (11.3–21.9)	0.443
RISK FACTORS								
Sufficient physical activity								
No activity	1036	530	51.2 (47.3–55.0)	361	34.9 (31.2–38.7)	144	13.9 (11.2–17.1)	0.005
Activity but not sufficient	1619	810	50.0 (46.7–53.3)	561	34.7 (31.5–38.0)	248	15.3 (12.9–18.1)	
Sufficient activity	2700	1567	58.0 (54.9–61.1)	813	30.1 (27.4–33.0)	320	11.8 (9.8–14.3)	
BMI								
Underweight	90	38	41.9 (30.3–54.4)	36	39.7 (27.6–53.2)	17	18.4 (10.5–30.2)	0.285
Normal	2019	1085	53.8 (50.3–57.2)	645	32 (28.8–35.3)	288	14.3 (11.6–17.4)	
Overweight	1744	1000	57.3 (54.0–60.6)	535	30.7 (27.7–33.8)	209	12.0 (9.8–14.6)	
Obese	1300	687	52.9 (48.9–56.8)	442	34.0 (30.3–37.9)	171	13.2 (10.8–16.0)	
Current smoker	752	313	41.6 (35.9–47.5)	263	34.9 (29.5–40.9)	176	23.5 (17.9–30.0)	<0.001
Alcohol related risk of harm								
Lifetime risk of alcohol-related harm	1901	926	48.7 (44.9–52.6)	685	36.0 (32.4–39.8)	290	15.3 (12.4–18.7)	<0.001
Risk of alcohol-related injury	736	334	45.4 (39.5–51.4)	291	39.5 (33.6–45.8)	111	15.1 (11.4–19.8)	0.008
Sufficient consumption of fruit and vegetable								
Neither sufficient	2870	1448	50.4 (47.6–53.3)	996	34.7 (32.0–37.4)	427	14.9 (12.8–17.3)	<0.001
Either suff fruit or veg	2222	1299	58.4 (55.5–61.4)	649	29.2 (26.6–32.0)	274	12.3 (10.4–14.7)	
Both suff fruit and veg	369	219	59.3 (52.8–65.5)	119	32.2 (26.3–38.8)	31	8.5 (5.7–12.4)	
OVERALL	5464	2968	54.3 (52.3–56.3)	1764	32.3 (30.5–34.2)	733	13.4 (12.0–15.0)	

Table 5 highlights the results of the multi-nominal modelling with higher odds ratios shown for all four social capital variables for the lowest level of wellbeing. These higher odds ratios remained even after adjusting for eight known confounders. The most marked increase in odds ratios were for the social capital variable assessing control over decisions that affect life. Those who do not have control were over 10 times more likely to have poor wellbeing.

**Table 5:** Multinomial logistic regressions of overall wellbeing by social capital indicators

	Unadjusted		Model 1		Model 2	
	OR (95% CI)	*P* value	OR (95% CI)	*P* value	OR (95% CI)	*P* value
Feeling that neighbourhood is a safe place						
Good wellbeing (reference)	1.00		1.00		1.00	
Scoring neither well or badly						
Yes (feel safe place)	1.00		1.00		1.00	
No, don’t know, not sure	2.51 (1.73–3.64)	<0.001	2.46 (1.70–3.56)	<0.001	2.12 (1.46–3.09)	<0.001
Poor wellbeing						
Yes (feel safe place)	1.00		1.00		1.00	
No, don’t know, not sure	3.41 (2.23–5.22)	<0.001	3.11 (2.00–4.83)	<0.001	2.54 (1.69–3.83)	<0.001
Neighbourhood people generally trust one another						
Good wellbeing (reference)	1.00		1.00		1.00	
Scoring neither well or badly						
Yes (trust one another)	1.00		1.00		1.00	
No, don’t know, not sure	1.73 (1.39–2.14)	<0.001	1.69 (1.36–2.10)	<0.001	1.52 (1.22–1.90)	<0.001
Poor wellbeing						
Yes (trust one another)	1.00		1.00		1.00	
No, don’t know, not sure	2.45 (1.79–3.34)	<0.001	2.35 (1.73–3.18)	<0.001	2.00 (1.45–2.76)	<0.001
Feeling safe in own home						
Good wellbeing (reference)	1.00		1.00		1.00	
Scoring neither well or badly						
All of the time (feel safe)	1.00		1.00		1.00	
Most, some or none of the time	2.11 (1.71–2.59)	<0.001	2.15 (1.75–2.65)	<0.001	2.10 (1.71–2.59)	<0.001
Poor wellbeing						
All of the time (feel safe)	1.00		1.00		1.00	
Most, some or none of the time	2.47 (1.86–3.28)	<0.001	2.47 (1.86–3.29)	<0.001	2.37 (1.76–3.19)	<0.001
Control over decisions affect life						
Good wellbeing (reference)	1.00		1.00		1.00	
Scoring neither well or badly						
Agree (have control over decisions)	1.00		1.00		1.00	
Neutral	2.30 (1.17–4.51)	0.016	2.39 (1.21–4.72)	0.012	2.12 (1.06–4.23)	0.034
Disagree	3.76 (2.16–6.54)	<0.001	3.71 (2.12–6.49)	<0.001	3.26 (1.86–5.72)	<0.001
Poor wellbeing						
Agree (have control over decisions)	1.00		1.00		1.00	
Neutral	4.22 (2.02–8.81)	<0.001	4.74 (2.28–9.86)	<0.001	4.12 (1.99–8.51)	<0.001
Disagree	11.58 (6.41–20.93)	<0.001	11.78 (6.69–20.76)	<0.001	9.81 (5.64–17.06)	<0.001

OR – odds ratio; CI – confidence interval

Model 1: adjusted by sex and age

Model 2: adjusted by sex, age and other socio-demographic and socio-economic indicators (country of birth, area of residence, educational attainment, marital status, money situation, number of adults)

## Discussion

This analysis has detailed the distribution of wellbeing in the South Australian adult population with high levels reported for females, older persons, those living in rural areas, married and those able to save. Social capital was associated with the three levels of wellbeing with, in all cases, worse measures of social capital indicating lower levels of wellbeing. When multi-nominal level logistic regression modelling were undertaken on the four social capital variables, in each instance the unadjusted, adjusted by age and sex, and the fully adjusted models, resulted in much higher odds ratios indicating that the relationship between low levels of social capital are associated with low levels of wellbeing in the South Australian community.

The current government of South Australia aims to become the first government in the world to systematically measure and build wellbeing across different cohorts and lifespans of the society to reduce the number of people experiencing catastrophic mental illness and to improve the resilience of the population. The analysis presented here goes some way in providing avenues for improved targeting at the broad population level.

If the aim of positive psychology is to ‘foster the factors that allow individuals, communities and societies to flourish’ [25], based on the results of this research, the incorporation of social capital as an important factor in the endeavour to increase wellbeing, is warranted. While previous interventions based on social capital have shown positive effects on wellbeing in selected groups [2, 26, 27], positive psychology research has not yet fully incorporated social capital as an important influence in understanding how individuals and communities cope in times of stress with social capital an ‘underutilized resource’ in determining and increasing resilience [7, 16]. It has been shown that social capital is at its strongest when disasters occur or when ‘conflict, problems or change’ are presented to communities [12]. Although much research focuses on physical/environmental disasters our results show that the close relationship between social capital and wellbeing in non-environmental emergency periods, indicates an investment in social capital could assist in increasing wellbeing. Considerable resources are often invested in physical infrastructure by governments, for example with stronger building codes in preparation of a natural disaster [7]. Social capital generated in non-physical emergency times with investment in non-physical aspects of our societies, can have beneficial long-term effects.

Somewhat surprising in our analysis was the lack of meaningful associations between the chronic diseases examined (except for current asthma) and the levels of wellbeing. Previous research has reported associations between positive wellbeing and a range of health outcomes including cardiovascular health [28]. A call for research into the association between wellbeing and risk factors is somewhat answered in this analysis with strong associations reported although our analysis was limited to only four risk factors [28]. Also called for, and not addressed in our research, is the role of positive health factors [28].

The strong relationship between social capital and wellbeing is not surprising given both are related to individuals and communities, each are seen as a resource or an asset for the other, both have similar pathways and relationships, both have similar confounding factors including socio-economic status, both can be invested in, both are open to development and both are measurable. Negative critiques of wellbeing often cite the one dimensional focus on the individual associated with resilience policy approaches [29]. The broadness of what is associated under the social capital mantel complements this limiting factor. As such, possible policy interventions such as strengthening social infrastructure and community resilience should also be implemented to assist in the desired increases of wellbeing in the community.

The weaknesses associated with the study include the cross-sectional nature of the data collection such that no cause and effect can be implicated. The mode of data collection, telephone, could also be a weakness with socially desirable responses possible, and low response rates resulting in bias estimates. In addition, contention still exists in terms of the lack of conceptual clarity of social capital [7] and the correct objective and subjective way to measure social capital in the population [1, 4, 11, 30]. A further weakness of our study is the limitation of the measuring of social capital to four questions. We acknowledge that our measure of social capital is a broad brush approach and not specifically encompassing the different types of social capital such as bonding, bridging and linking [4, 10]. We also acknowledge that our wellbeing questions are somewhat limited in scope, limited by the time on the telephone, and that well-developed wellbeing-related questionnaires exist [31–33].

Notwithstanding, the strengths of this study include the large sample size, the representative population and the value of adding, as called for by others, broad population research in the positive psychology and wellbeing arenas [14, 33]. Also a strength is the use of an extensive list of confounders in the multivariable analyses. As highlighted by Harphan et al. [9], the desired confounders that should be incorporated into any social capital analysis include socio-economic status, education, gender and number of people per household all of which we have adjusted for in our analysis. The use of an on-going surveillance system as the collection mode, with consistent use of questions and methods, will allow for population groups to be monitored over time and evaluations to be assessed within the population and priority groups.

## Conclusion

This research has highlighted the relationship between wellbeing, resilience and social capital showing how inter-related they are, how important the associations are and highlighting areas for possible increased policy focus. As argued by Bernier and Meinzen-Dick [16], this relationship has been underexplored. The positive wellbeing attributes of individuals and their relationship to others in their community are important considerations. The work being undertaken in South Australia to improve individual and community wellbeing will continue to be evaluated so that the value of prevention rather that treatment can be assessed.

## References

[1] Berry HL, Welsh JA (2010). Social capital and health in Australia: an overview from the household, income and labour dynamics in Australia survey. Soc Sci Med.

[2] Bolier B, Haverman M, Westerhof GJ, Riper H, Smit F, Bohlmeijer E (2013). Positive psychology interventions: a meta-analysis of randomized controlled studies. BMC Public Health.

[3] Mitchell J, Vella-Brodrick D, Klein B (2010). Positive psychology and the internet: a mental health opportunity. Electronic Journal of Applied Psychology.

[4] Almedon AM (2005). Social capital and mental health: an interdisciplinary review of primary evidence. Soc Sci Med.

[5] Bonanno GA, Westphal M, Mancini AD (2011). Resilience to loss and potential trauma. Annu Rev Clin Psychol.

[6] Cornum R, Matthews MD, Seligman ME (2011). Comprehensive soldier fitness: building resilience in a challenging institutional context. Am Psychol.

[7] Aldrich DP, Meyer MA (2015). Social capital and community resilience. Am Behav Sci.

[8] Seligman ME (2013). Building the state of wellbeing: a strategy for South Australia.

[9] Harphan T, Grant E, Thomas E (2002). Measuring social capital within health surveys: key issues. Health Policy & Planning.

[10] Poortinga W, Suffolk C. (2011). Community resilience and wellbeing in Wales: A secondary analysis of the 2007 and 2009 citizenship survey. Cardiff University. Downloaded from ???

[11] Szreter S, Woolcock M (2003). Health by association? Social capital, social theory and the political economy of public health. Int J Epidemiol.

[12] Australian Bureau of Statistics (ABS). (2002). Social capital and social wellbeing. Discussion Paper. Commonwealth of Australia. Downloaded from http://www.abs.gov.au/websitedbs/D3110122.NSF/6ead87599a9a3b0bca256eaf00836eb9/150bcb152250ddcfca256c220080ba47!OpenDocument

[13] Taylor A, Dal GE (2008). Chronic disease and risk factor surveillance using the SA Monitoring and surveillance system (SAMSS) - history, results and future challenges. Public Health Bull South Aust.

[14] Kobau R, Seligman ME, Peterson C, Diener E, Zack MM, Chapman D, Thompson W (2011). Mental health promotion in public health: perspectives and strategies from positive psychology. Am J Public Health.

[15] Seligman ME, Park N, Steen TA (2004). A balanced psychology and a full life. Philosophical Transactions of the Royal Society London.

[16] Bernier Q, Meinzen-Dick R (2014). Resilience and social capital.

[17] Population Research and Outcome Studies (PROS) (2004). South Australian Monitoring and surveillance system (SAMSS) technical report. Report 1/04, 2004: survey methodology.

[18] American Association for Public Opinion Research (AAPOR). (2011). Standard definitions: final dispositions of case codes and Outcome rates for surveys, 7th Edition. 7th edition ed. Deerfield, Illinois, USA: AAPOR; 2011.

[19] Australian Government. Australia's physical activity and sedentary behaviour guidelines. Department of Health and Aged Care. Canberra; 2005. Available at http://www.health.gov.au/internet/main/publishing.nsf/Content/health-pubhlth-strateg-phys-act-guidelines.

[20] World Health Organization (WHO). BMI classification. Geneva, Switzerland; 2010. Available from: http://www.who.int/nutrition/publications/obesity/WHO_TRS_894/en/.

[21] NHMRC (National Health and Medical Research Council). Australian Guidelines to reduce health risks from drinking alcohol Commonwealth of Australia; 2009. Available at https://www.nhmrc.gov.au/guidelines-publications/ds10.

[22] NHMRC (National Health and Medical Research Council). The dietary Guidelines for Australians; 2005. Downloaded from https://www.nhmrc.gov.au/guidelinespublications/n29-n30-n31-n32-n33-n34.

[23] Evans J, Macrory I, Randall C (2015). Measuring National Well-Being: life in the UK, 2015: Office for National Statistics.

[24] Abdallah S, Shah S (2012). Well-being patterns uncovered: an analysis of UK data.

[25] Fredrickson BL (2001). The Role of Positive Emotions in Positive Psychology. The Broaden-and-Build Theory of Positive Emotions. Am Psychol.

[26] Mongrain M, Anselmo-Matthews T (2012). Do Positive Psychology Exercises Work? A Replication of Seligman et al. J Clin Psychol.

[27] Seligman MEP, Steen TA (2005). Positive Psychology Progress. Empirical Validation of Interventions. Am Psychol.

[28] Boehm JK, Kubzansky LD (2012). The heart's content: the association between positive psychological wellbeing and cardiovascular health. Psychol Bull.

[29] Allmark P, Bhanbhro S, Chrisp T (2014). An argument against the focus on Community Resilience in Public Health. BMC Public Health.

[30] Altschuler A, Somkin CP, Adler NE (2004). Local services and amenities, neighborhood social capital, and health. Soc Sci Med.

[31] Kern ML, Waters LE, Adlea A, White MA (2015). A multidimensional approach to measuring wellbeing in students: Application of the PERMA framework. J Posit Psychol.

[32] Forgeard MJC, Jayawickreme E, Kern ML, Seligman MEP (2001). Doing the right thing: Measuring wellbeing for public policy. Int J Wellbeing.

[33] OECD. OECD Guidelines on measuring subjective wellbeing. OECD Publishing; 2013. ISBN 978-92-64-19165-5. 10.1787/9789264191655-en.

